# Clinical outcomes and treatments effectiveness in status epilepticus resolved by antiepileptic drugs: A five‐year observational study

**DOI:** 10.1002/epi4.12383

**Published:** 2020-03-02

**Authors:** Niccolò Orlandi, Giada Giovannini, Jessica Rossi, Maria Cristina Cioclu, Stefano Meletti

**Affiliations:** ^1^ Neurology Unit OCB Hospital Azienda Ospedaliera‐Universitaria Modena Italy; ^2^ Department of Biomedical, Metabolic and Neural Science University of Modena and Reggio Emilia Modena Italy

**Keywords:** lacosamide, levetiracetam, mortality, outcomes, status epilepticus, valproate

## Abstract

**Objective:**

To evaluate clinical outcomes and treatment effectiveness of status epilepticus finally resolved by nonbenzodiazepine antiepileptic drugs (AEDs).

**Methods:**

All consecutive SE episodes observed from September 1, 2013, to September 1, 2018, and resolved by AEDs were considered. Diagnosis and classification of SE followed the 2015 ILAE proposal. Nonconvulsive status (NCSE) diagnosis was confirmed according to the Salzburg EEG criteria. The modified Rankin Scale and deaths at 30 days from onset were used to evaluate outcomes.

**Results:**

A total of 277 status episodes (mean age 71 years; 61% female) were treated and resolved by antiepileptic drugs after 382 treatment trials. 68% of the SE resolved after AED use as first/second treatment line, while subsequent trials with AEDs gave an additional 32% resolution. A return to baseline conditions was observed in 48% of the patients, while overall mortality was 19% without significant changes across the study years. Mortality was higher in NCSE than in convulsive SE (22.5% vs 12.9%; *P* < .05), while mortality did not differ in SE episodes resolved by a first/second AED trial (17.2%) versus SE resolved by successive treatment trials (18.9%). The resolution rate of intravenous AEDs was 82% for valproate, 77% for lacosamide, 71% for phenytoin, and 62% for levetiracetam. No significant differences were found in head‐to‐head comparison, but for the valproate‐levetiracetam one that was related to NCSE episodes in which valproate resulted to be effective in 86% of the trials while levetiracetam in 62% (*P* < .002).

**Significance:**

A high short‐term mortality, stable over time, was observed in SE despite resolution of seizures, especially in SE with nonconvulsive semiology. Comparative AED efficacy showed no significant differences except for higher resolution rate for valproate versus levetiracetam in NCSE.

**Key Points**

- Short‐term mortality in established SE over a five‐year period was about 20% despite status resolution by nonbenzodiazepine AEDs
- Mortality was strongly related to SE semiology, being 23% in nonconvulsive SE compared with 12% in SE with prominent motor features
- Mortality was not related to SE resolution after a first AED trial compared with more later resolutions, after subsequent AED trials
- Valproate showed higher efficacy (86%) than levetiracetam (62%) in nonconvulsive SE episodes

## 1 INTRODUCTION

Status epilepticus (SE) is a condition resulting either from the failure of the mechanisms responsible for seizure termination or from the initiation of mechanisms which can lead to abnormally prolonged seizures.^1^ Besides stroke, it represents one of the most important neurological emergencies and a life‐threatening condition, with an important mortality and morbidity.^2^, ^3^, ^4^, ^5^ A prompt and appropriate treatment is urgently needed to prevent brain damage, systemic consequences, and poor outcomes.^6^, ^7^, ^8^

Current protocols are based on a three‐stage approach, according to which benzodiazepines are used as first‐line agents. Indeed, randomized controlled trials (RCTs) have demonstrated that intravenous lorazepam^9^, ^10^ or intramuscular midazolam^11^ is the most efficient option in early status. After their failure, SE is considered to be established (ESE), requiring the administration of antiepileptic drugs (AEDs) given intravenously. In refractory cases, the use of anesthetics and coma induction are requested. However, despite an increasing knowledge regarding the pathophysiology and molecular mechanisms taking place during SE,^12^ its treatment, especially in the late stages, is still controversial as well as the impact of therapeutic coma on survival.^13^, ^14^ For these reasons, it is important to optimize SE treatment in order to avoid, when possible, anesthetic use and intensive care unit (ICU) admission.

For years, phenytoin (PHT) and phenobarbital (PB) have been used in patients with SE refractory to benzodiazepines, despite their adverse events profile as well as the absence of a clear superiority toward other drugs, in particular valproate.^15^, ^16^, ^17^, ^18^ Due to the increasing number of AEDs on the market, a growing trend in the prescription of newer AEDs in SE over the last decade has been reported.^19^ However, improved clinical outcomes over time in SE have not been demonstrated. Indeed, at least in the study of Beuchat et al^19^ a higher risk of SE refractoriness and new disability at hospital discharge was observed in more recent years, when newer AEDs have been increasingly used compared to previous years. Notably, considering the few high‐class RCTs, clinical practice is still influenced by experts' opinions, and while clinical guidelines emphasize the need for rapid control of benzodiazepine‐resistant SE, they do not provide guidance regarding the choice of medication on the basis of either efficacy or safety.^20^, ^21^, ^22^, ^23^, ^24^ To fill this gap, very recently the results of the Established Status Epilepticus Treatment Trial (ESETT), a randomized, blinded, adaptive trial that compared the efficacy and safety of levetiracetam, fosphenytoin, and valproate in children and adults with benzodiazepine‐refractory convulsive status epilepticus, have been published.^25^ The three study drugs showed the same efficacy leading to seizure cessation at 60 minutes in about half of the patients. However, while almost all RCTs on benzodiazepine‐resistant SE are focused on status with convulsive semiology, nonconvulsive forms of SE (NCSE) are equally frequent and now increasingly recognized in clinical practice.^5^, ^26^

In this study, we evaluated the clinical outcomes and treatment effectiveness of nonbenzodiazepine intravenous antiepileptic drugs (AEDs) in a cohort of consecutive status epilepticus episodes that were finally resolved by AEDs.

## 2 METHODS

### 2.1 Patients and adopted definitions

All consecutive SE episodes prospectively registered at the Ospedale Civile Baggiovara, the hub for neurological emergency of the Modena District in northern Italy, occurring in adolescents and adults (≥14 years) and observed from September 1, 2013, to September 1, 2018, were reviewed.

Before 2015, we adopted an operational definition of SE that was defined as a continuous seizure that lasts ≥5 minutes or two or more discrete seizures between which there is not a complete recovery of consciousness.^27^ After 2015, the operational definition of SE proposed by Trinka et al was adopted and prospectively applied.^1^ Thus, relying on evidences from animal studies, the time point to define a SE has been set to 5 minutes for tonic‐clonic SE, 10 minutes for focal SE with impaired consciousness, and 10‐15 minutes for absence status epilepticus.

In cases of SE without prominent motor semiology, the diagnosis of nonconvulsive status epilepticus (NCSE) was reviewed according to the Salzburg EEG criteria.^28^, ^29^ SE in the context of postanoxic episodes were excluded from the study.

According to treatment outcomes, established status epilepticus was defined as a SE without clinical and/or electroencephalographic resolution after the administration of first‐line agents (benzodiazepines). Refractory status epilepticus was defined as a SE that persists, regardless of the delay since the onset of the seizure, after failure of a trial of at least one AED, at adequate dosage, requiring consequently the use of anesthetic drugs.

According to the 2015 proposed classification of SE,^1^ etiology was defined as acute symptomatic, remote symptomatic, and progressive symptomatic. When it was not possible to identify a clear etiology, SE was classified as unknown. An idiopathic category was adopted, even if it was excluded from the recent classification, for certain focal or generalized epileptic syndromes with specific clinical and EEG characteristics. Moreover, SE was classified as multifactorial when more than one of the aforementioned categories was simultaneously present and judged equally important in SE determination.

### 2.2 Procedures

As reported in previous studies by our group,^4^, ^26^ a specific Status Epilepticus Form was used to collect prospectively, for each case, the following information: age; gender; place of residence; site and date of SE observation and date of SE onset; history of epilepsy prior to SE; comorbidities; level of disability before SE (using the modified Rankin Scale; mRS); level of consciousness at first medical evaluation (using the Glasgow Coma Scale; GCS); etiology; semiology of SE before treatment; type and results of neuroradiologic studies; type, duration, and dosage of AED; anesthetic drugs; and other therapies used.

The form was filled in by the first doctor who took care of the patient (in all cases, a neurologist or a neurointensivist) or by the staff of the neurophysiology unit who performed the first EEG examination of a suspected SE case. It is worth noting that a neurology ward serves the hospital 24 h/d for 7 d/wk, and the same neurophysiology staff records all the EEGs.

A complete electrolytic, metabolic, and hematologic workup was performed for every single patient, whereas brain imaging (CT scan or MRI) and lumbar puncture were performed as needed. At least one EEG recording of variable duration according to clinical needs was obtained for each patient.

A neurologist, trained in epilepsy, then revised all the forms and the EEG interpretation and completed any missing information consulting the hospital Informatics Database. As an additional quality control of the study protocol, we also checked all the patients discharged from the hospital in the analyzed years with an “epilepsy” or “seizure” ICD‐9 discharge code.

### 2.3 Study outcomes

All outcomes measures were calculated on SE episodes that were finally resolved by AEDs, therefore not requiring an escalation strategy to anesthetics and intensive care unit (ICU) admission.

The primary outcomes of the study were as follows: (a) the efficacy of the AEDs administered to stop SE; and (b) 30‐day mortality and disability through the application of the mRS.

We considered a trial with one specific AED a success in stopping SE when (a) the AED was the last drug administered within 72 hours prior to the clinical and/or EEG resolution of SE and (b) the SE did not recur during the entire hospital observation of the patient.^30^

For SE episodes with multiple AED trials, treatment schedules for dose adjustment of concomitant medication were reviewed.

Secondary outcomes were the incidence and features of adverse events observed for each drug. We classified an event as “adverse event” if it appears in close temporal relation to the administration of the drug and if it is reported in the safety profile of the drug.

### 2.4 Statistical analysis

The statistical analysis was performed using SPSS software (version 26). Descriptive statistics was used for the evaluation of demographic and clinical data. In addition, we calculated 95% confidence intervals (95% CIs) for both mortality and SE resolution rate of the different AEDs. Categorical variables were compared using the Pearson χ^2^ test or the Fisher exact test when required. The statistical significance cutoff was set at 0.05. Adjustment for multiple comparisons (Bonferroni) was used to test for comparative AED efficacy (among VPA, LCM, PHT, and LEV) with α = 0.0125.

### 2.5 Standard protocol approvals, registrations, and patient consents

The scientific advisory boards of our institution approved the research protocol according to local regulations and the local ethics committee approved the retrospective analysis of patients' data. Treatment choices and iv AED doses were performed following an internal treatment protocol based on the recommendations of international guidelines^20^, ^21^, ^22^ (publicly available at http://salute.regione.emilia-romagna.it/percorso-epilessia/PDTASE_AOU.pdf). Table S1 shows the AED doses (bolus and maintenance) used. Table S2 shows the data concordant to the STROBE statement.

## 3 RESULTS

**Figure 1:**
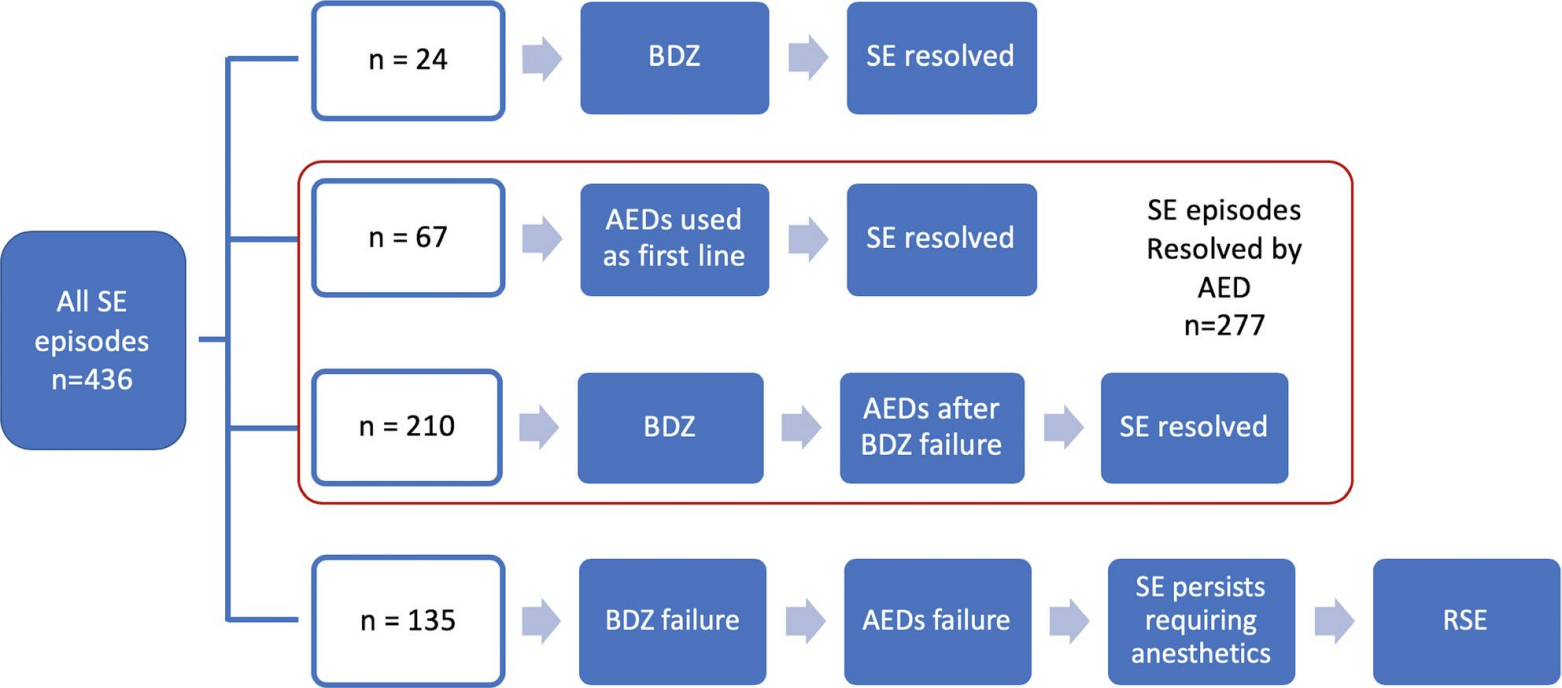
Study flowchart. Out of the 436 status episodes (SE), 24 were resolved after intravenous benzodiazepine administration and were not further analyzed (first row), as well as 135 SE episodes (31%) that were treated with anesthetics after subsequent failures of benzodiazepines and one or more antiepileptic drugs (AEDs) (last row). Overall, 277 SE episodes were resolved by AEDs (63.5%) (red box): In 67 cases (15%), AEDs were used as first‐line agent, while in 210 episodes, AEDs were used after benzodiazepines failure

We observed 436 episodes of SE (mean age 70 years; 59% female). Figure 1 shows the study flowchart and the final population of SE that were resolved by AEDs (64% of the total; 277 episodes).

Of the 277 episodes of SE resolved by AEDs, 170 (61%) occurred in female patients. The age of the patients ranged from 14 to 94 years (mean: 70.2 years; median: 75 years). With the exception of six patients (three African, two Asian, and one Hispanic), all the episodes occurred in Caucasian patients. Demographic and clinical variables of the final study population are reported in Table 1.

**Table 1:** Demographics, clinical features, and etiology relative to 277 status episodes resolved by antiepileptic drugs

	n	%
Total	277	100%
Gender		
Female	170	61%
Age		
Range (years)	14‐98	
Mean (years)	71.1	
Onset in hospital	94	34%
Previous history of epilepsy	80	29%
Causes		
Cerebrovascular	78	28%
Brain tumors	31	11%
Meningoencephalitis	11	4%
Sepsis	18	6%
Metabolic dysregulation	20	8%
Inducing factors in epilepsy	40	14%
Toxic	7	3%
Multifactorial	34	12%
Brain inflammation	9	4%
Trauma	6	2%
Epileptic encephalopathy	3	1%
Unknown	16	6%
Others	4	1%
Etiology		
Acute symptomatic	142	51%
Remote symptomatic	49	18%
Progressive symptomatic	40	14%
Multifactorial	28	10%
Unknown	16	6%
Idiopathic	2	1%
Semiology		
Prominent motor ‐ CSE	139	50%
GCSE	19	7%
GCSE‐>NCSE	31	11%
FMSE	51	18%
FMSE‐>NCSE	35	13%
MSE	0	0%
MSE‐>NCSE	3	1%
NCSE	138	50%
Outcomes (30 d)		
Return to baseline conditions	127	48%
Mortality	52	19%

Abbreviations: CSE, Convulsive status epilepticus; FMSE, focal motor status; GCSE, generalized convulsive status; MSE, myoclonic status epilepticus; NCSE, nonconvulsive status epilepticus.

According to the prevalent seizure type and EEG features, we observed 138 episodes (50%) of NCSE, 51 cases (18%) with focal motor status (FMSE), 19 (7%) with generalized convulsive status (GCSE), and three with myoclonic status epilepticus (MSE) evolving into NCSE (1%). Moreover, we observed 31 episodes of GCSE and 35 of FMSE with evolution into NCSE.

### 3.1 Treatment choices and SE resolutions

Overall, 382 AED trials were performed in the 277 SE finally resolved by AEDs.

Considering the different AEDs, VPA and LEV were the most frequently used AEDs as they were prescribed in nearly half of the episodes. PHT and LCM were used in 55 and in 40 trials, respectively. PB was prescribed rarely (6 episodes, always after the failure of at least other two AEDs), and in 17 cases, other compounds were administered by enteral route: perampanel, carbamazepine/oxcarbazepine, topiramate, and pregabalin. These last drugs were not further considered for statistical comparisons. The prescription trends of PHT and VPA and of the newer AEDs LEV and LCM are reported in Figure S1. While prescriptions of VPA and LEV were approximately constant during the five‐year study period, LCM prescriptions gradually increased, while PHT decreased.

**Figure 2:**
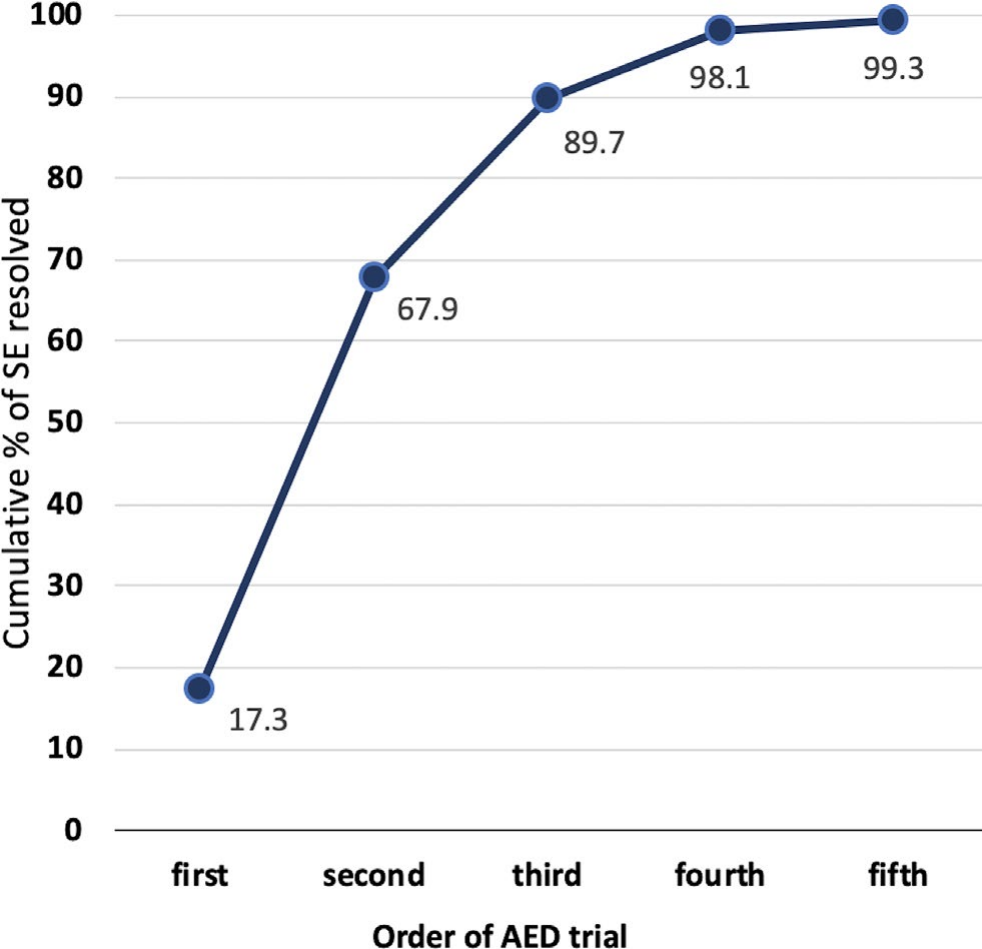
Cumulative resolutions according to treatment trials. Cumulative percentage of status epilepticus resolution as a function of subsequent trials with antiepileptic drugs

Considering the resolution rate according the order of AED administration, intravenous AEDs were used as first‐line treatment in 65 SE episodes in which the treating doctor had either concerns about respiratory failures or in case of patients with a previous history of epilepsy and SE due to rapid withdrawal of AED (ie, valproate, or levetiracetam). The overall success rate in these episodes was 74% (48/65). AEDs were used as second‐line drugs in 194 trials after failure of benzodiazepines (n = 177) or failure of a previous AED (n = 17) with a success rate of 68% (132/194). Finally, an iv AED was used in selected patients as third‐, fourth‐, or fifth‐line agent, after a previous AED failure, in 72, 25, and 3 trials, respectively. In these cases, the treating physician preferred to avoid an escalation strategy to anesthetics and ICU admission. This choice could be possible in light of the semiology of this subgroup of SE. Indeed, only the 7% of these SE episodes were represented by convulsive status epilepticus: The 37% were NCSE since onset or motor SE evolving to a NCSE (37%), or focal motor SE (18%). Considering the cumulative percentage of SE resolved by subsequent AED trials, Figure 2 shows that the cumulative percentage of SE resolution was 68% after a first/second AED trial. After that, a trial of a third (or more) AED added about a 30% probability of success. To check for possible dose adjustments of a previously administered AED, we reviewed all the case report forms observing modifications in concomitant medications in only six out of 117 SE episodes treated by multiple iv AED trials. Bolus and maintenance doses in this group were “standard” doses as recommended in the internal treatment protocol (see the Methods section).

The response rates of single AEDs are reported in Table 2. No significant difference was appreciated in any head‐to‐head comparison with the exception of the VPA‐LEV comparison showing that VPA had a higher resolution rate (82%) than LEV (62%) (*P* < .005). The clinical and demographic features and the order of the AED administration in the patient groups exposed to the valproate and levetiracetam are reported in Table 3. No differences in demographics, etiologies, or order of AED administration were found except for trend in a slightly higher proportion of SE with nonconvulsive semiology in the SE episodes treated with VPA. Evaluating the efficacy of VPA and LEV according to SE semiology (Table 4), we observed a significantly higher response rate in the VPA‐treated relative to the LEV‐treated group in status with nonconvulsive semiology (*P* < .002).

**Table 2:** Antiepileptic drugs used and rate of status epilepticus resolutions

	No. of trials	No. of SE resolved	% resolution by treatment trial	95% CI
Total treatment trials	382	277	73%	70‐75
Valproate	139	114	82%	77‐89
Levetiracetam	125	78	62%	54‐71
Phenytoin	55	39	71%	59‐83
Lacosamide	40	31	77%	65‐91
Phenobarbital	6	5	83%	n.c.
Other non‐iv AEDs^a^	17	10	58%	n.c.

Abbreviation: n.c., not calculated.

^a^ Enteral use of perampanel, carbamazepine/oxcarbazepine, topiramate, and pregabalin.

**Table 3:** Clinical features of SE treated with valproate (VPA) and levetiracetam (LEV)

	VPA (n = 139)		LEV (n = 125)		*P*	
	n	%	n	%		
Gender						
Female	89	64%	77	62%	.68	χ^2^
Age						
Range (years)	16‐94	14‐93				
Mean (years)	72		71.5		.76	U
Onset in hospital	48	35%	45	36%	.80	χ^2^
Previous history of epilepsy	37	27%	41	33%	.27	χ^2^
AED used						
As first line	28	21%	27	21%	.66	χ^2^
As second line	77	55%	78	62%	.20	χ^2^
As third or more	34	24%	20	16%	.08	χ^2^
Causes						
Cerebrovascular	43	31%	35	28%	.88	χ^2^
Brain tumors	12	9%	18	14%	.14	χ^2^
Meningoencephalitis	4	3%	7	6%	.37	f
Sepsis	11	8%	7	6%	.48	f
Metabolic dysregulation	14	10%	7	6%	.25	f
Inducing factors in epilepsy	17	12%	19	15%	.38	χ^2^
Toxic	5	4%	3	2%	.73	f
Multifactorial	17	12%	15	12%	.82	χ^2^
Brain inflammation	4	3%	4	3%	1	F
Trauma	2	1%	5	4%	.26	f
Epileptic encephalopathy	1	1%	1	1%	1	f
Unknown	6	4%	3	2%	.51	f
Others	3	2%	1	1%	.62	f
Etiology						
Acute symptomatic	73	53%	66	53%	.96	χ^2^
Remote symptomatic	28	20%	21	17%	.49	χ^2^
Progressive symptomatic	15	11%	23	18%	.79	χ^2^
Multifactorial	16	12%	10	8%	.34	χ^2^
Unknown	6	4%	4	3%	.75	f
Idiopathic	1	1%	1	1%	1	f
Semeiology						
Prominent motor – CSE	60	43%	69	55%	.06	χ^2^
GCSE	9	7%	9	7%	1	f
GCSE‐>NCSE	14	10%	15	12%	.62	χ^2^
FMSE	17	12%	23	18%	.16	χ^2^
FMSE‐>NCSE	17	12%	22	18%	.22	χ^2^
MSE	3	2%	0	0%	.24	f
NCSE	79	57%	56	45%	.06	χ^2^
Outcomes (30 d)						
Return to baseline conditions	53	39%	60	50%	.08	χ^2^
Mortality	32	23%	22	18%	.31	χ^2^

Abbreviations: CSE, Convulsive status epilepticus; FMSE, focal motor status; GCSE, generalized convulsive status; MSE, myoclonic status epilepticus; NCSE, nonconvulsive status epilepticus.

**Table 4:** Success rate of valproate and levetiracetam in resolving status epilepticus with prominent motor and nonmotor semiology

	Valproate			Levetiracetam			
	No. of trials	Success rate	95% CIs	No. of trials	Success rate	95% CIs	*P*
In prominent motor SE	60	76.7%	71‐82	69	62.3%	56‐68	.08
In nonconvulsive SE	79	86.1%	79‐94	56	62.5%	49‐75	.002

### 3.2 Safety

No severe adverse events were observed. Details of adverse events are reported in Table S3. LCM and LEV showed the lowest occurrence of adverse events (2.4%), whereas VPA administration was characterized by the highest incidence of adverse events (9.4%). Head‐to‐head comparison between AEDs showed that LEV had a significantly lower incidence of adverse events when compared to PHT (*P* < .01) and VPA (*P* = .02). Head‐to‐head comparisons between LCM, PHT, and VPA did not show any significant difference. As regards traditional AEDs as a group (PHT, VPA, and PB), they showed a higher incidence of adverse events (10%) when compared to newer agents (LEV and LCM) (3.6%) (*P* = .02).

### 3.3 Mortality and disability

Thirty‐day mortality was 19% (52/277; 95% CIs 17‐21). Mortality for each year of the study period was 22%, 16%, 18%, 27%, and 20% (no significant differences across study years). Mortality in NCSE cases was 22.5% (31/138; 95% CIs 19‐26), while that of convulsive forms was 12.9% (18/139; 95% CIs 10‐16) (*P* < .05). Mortality in SE episodes resolved by a first/second AED trial (17.2%; 95% CIs 15‐20) did not differ from mortality in SE resolved by successive AED trials (18.9% 95% CIs 14‐23) (*P* = .75). No significant difference in case fatality was observed in valproate‐ or levetiracetam‐treated SE episodes (Table 3). Finally, according to the etiology classification, unknown/de novo and acute symptomatic SE were associated with high short‐term mortality (see Table 5).

**Table 5:** Thirty‐day mortality rate according to SE etiology classification

	30‐d mortality (n)	Total episodes (n)	Mortality (%)	95% CI
Unknown	8	16	50%	n.c.
Acute symptomatic	29	142	20%	13‐27
Remote symptomatic	9	49	18%	7‐29
Multifactorial	3	28	11%	n.c.
Progressive symptomatic	3	40	8%	n.c.
Idiopathic	0	2	0%	n.c.

As regards disability, in nearly half of the cases a complete return to baseline conditions at 30 days from SE onset was possible (48%). In 145 cases (53%), there was a moderate‐severe disability (mRS > 3) at 30 days from the event.

## 4 DISCUSSION

In this study, we evaluated the clinical outcomes and effectiveness of antiepileptic drugs in status epilepticus in clinical practice over a five‐year period (2013‐2018). We limited our analysis to the pool of SE episodes that were, at the end, finally resolved by nonbenzodiazepine AEDs. In other words, we did not evaluate the SE episodes that required anesthetics and coma induction during status management. This was a deliberate choice that is justified by two main reasons. First, in this way we had a fixed final outcome: We analyzed a pool of SE that were resolved by AEDs. Second, we kept more constant several clinical variables that are often different in established SE compared to refractory and superrefractory SE.^26^, ^31^, ^32^, ^33^

Starting from the initial pool of 436 SE episodes, more than half (277; 64%) showed an electroclinical resolution after the administration of one or more AEDs. While in the majority of SE episodes, seizures stopped after the use of a drug as first/second treatment line, there was also a significant proportion of SE episodes for which multiple drug trials were performed before SE resolution. This means that even if a first or second AED trial was a treatment failure, there was still a 30% probability to stop seizures when an AED was used as a third or fourth treatment line. This finding is relevant, since it tells us that in SE episode in which it is reasonable to postpone anesthetic use and coma induction after the failure of benzodiazepines and/or of a first AED trial, there is still a significant probability to stop SE with a subsequent AED trial.

### 4.1 Comparative efficacy and safety of AEDs

In recent years, the question of whether newer AEDs are “better” than traditional agents in SE treatment has gained huge interest. To date, however, despite an increasing interest and prescriptions of newer AEDs, increased efficacy has not been demonstrated compared to the traditional ones.^19^, ^34^ Our data support these findings. Indeed, we observed an overall higher efficacy of valproate relative to levetiracetam, which was driven by a higher VPA response rates in SE episodes with nonconvulsive semiology. Notably, the two populations exposed to VPA and LEV were strikingly similar according to several demographic and clinical features and order of AED administrations, limiting several possible biases that are inherently present in a real‐life setting. Considering that levetiracetam is currently one of the mostly prescribed AEDs to treat nonconvulsive SE, our results make a cautionary note on this practice, especially in cases when VPA can be safely used without major concerns (ie, liver disease, mitochondrial disorders). To date, there are no RCTs focused on VPA‐LEV comparison in NCSE treatment. The ESETT trial as well as previous meta‐analyses did not find significant differences in efficacy between valproate and levetiracetam in benzodiazepine‐resistant convulsive status epilepticus.^16^, ^25^, ^35^ In contrast, our findings are similar to the results reported by Alvarez et al^36^ who found out that LEV was characterized by a higher incidence of failures (48.3%) than VPA (25.4%). Of course, it is impossible to directly compare our results with these studies, mainly because our analysis was—per definition—limited to SE episodes finally resolved by AEDs. However, a relevant issue is open by these results, especially relative to NCSE, that we think need to be evaluated by future studies.^37^ A speculative hypothesis to explain the high success rate of valproate in NCSE could be based on the pathophysiological mechanisms of nonconvulsive SE. Indeed, NCSE is often characterized by periodic EEG activities and rhythmic spike/sharp waves and slow waves, which we know reflect oscillatory mechanisms at thalamocortical level. One prototype of these types of activities is represented by the “absence status” observed in idiopathic (genetic) generalized epilepsies. Not surprisingly, historically, the drug of choice in this type of NCSE is VPA. Obviously, this speculation should be verified. For example, it could be very interesting in future studies to analyze the drug responsiveness according to the EEG pattern that characterizes the SE, and in particular the nonconvulsive status epilepticus.

Finally, it is interesting to remind that in some episodes (17 cases), other non‐iv AEDs have been used in status epilepticus as third‐/fourth‐line option, such as topiramate and perampanel. In some of them (10 episodes overall), an electroclinical resolution of SE was obtained. Recently, some studies have evaluated the possible effects of the administration of these drugs during SE.^38^ These drugs could be potentially used in selected patients to avoid sedation and its possible consequences,^14^, ^39^ but our data were too limited for making any considerations.

As regards safety, we did not observe severe adverse events in our clinical practice. PHT and VPA showed the highest incidence of adverse events, 13.2% and 9%, respectively, whereas LEV showed the lowest (2%). Apart from LEV, LCM was the AED with the best safety profile, and several studies in the literature have highlighted their excellent tolerability. Our data support these observations.

### 4.2 Clinical outcomes

We observed a relatively high short‐term mortality, about 19%, that was stable during the study period, although all the SE episodes were resolved by treatment. SE mortality rate has been previously reported to be between 9% and 39%.^33^, ^40^ Some studies have highlighted a reduction in mortality in the past decade, hypothesizing that this could be a consequence of a prompter recognition of nonconvulsive SE and an earlier, more aggressive, and more effective treatment.^41^, ^42^ Conversely, other studies have found a stable mortality rate^43^ and even an increasing number of patients with new morbidity at hospital discharge.^19^, ^44^ On the one hand, our results showed that mortality was not related to SE resolution after a first AED trial compared with more later resolutions, after subsequent AED trials. On the other hand, mortality was strongly related to nonconvulsive SE semiology (23% in NCSE versus 12% in convulsive SE), confirming recent findings by Leitinger et al,^5^ who observed that case fatality rates for NCSE and SE with prominent motor phenomena were 27.6% and 10.6%, respectively. Considering that in our case mix NCSE episodes were frequently observed, representing the half of all the SE episodes treated in the five‐year study period, we believe that the overall 20% case fatality can be explained according to the fact that nonconvulsive semiology is more often related to potentially severe SE etiologies (especially in comatose patients).

### 4.3 Study limitations

This is an observational study in which treatment outcomes were reviewed retrospectively. Even if the SE episodes were prospectively registered and treated according to a uniform protocol for SE, the study reflects the clinical practice and considerations of the treating physician at the time of SE observation. A possible concern is represented by the efficacy criteria that were adopted in our analysis. In the literature, several criteria that can drastically influence the results of observational studies have been reported.^30^ However, we believe that the criteria chosen were rigorous and, importantly, their evaluation was feasible for all the patients. Another potential important limit is that in SE episodes in which multiple drug trials are administered, each AED used could “benefit” from the therapeutic effect of the previously used AED. This could be especially true for AEDs with a different mechanism of action. Moreover, data about exact timing of AED delivery were missing, and only the sequence of drug administration was available. Dose changes in concomitant medications, another source of possible bias, were very rarely observed in our population. Finally, even if a standard protocol for SE treatment was defined in our institution, deviation in infusion rates is possible. However, even if we did not assess treatment adequacy with respect to guidelines, this does not seem to influence clinical outcomes.^45^

With regard to the generalization of the results obtained, the patients studied reflect the case mix of an academic hospital, a provincial reference center for all neurological emergencies. It is possible that the observed SE therefore reflects a selection of patients with severe etiologies, with a high percentage of SE with intrahospital onset and nonconvulsive semiology. Therefore, the observed data reflect and are comparable with cohorts with these characteristics.

## CONFLICT OF INTEREST

Dr Orlandi, Giovannini, Rossi, and Cioclu report no disclosure; Prof. Meletti received research grant support from the Ministry of Health (MOH) and from the nonprofit organization foundation "Fondazione Cassa di Risparmio di Modena—FCRM"; and has received personal compensation as scientific advisory board member for UCB and EISAI. We confirm that we have read the Journal's position on issues involved in ethical publication and affirm that this report is consistent with those guidelines.

## Supporting information

FigureS1

TableS1

TableS2

TableS3

## Data Availability

The authors state that the anonymized data on which the article is based will be shared by request of any qualified investigator.
