# Supplementary figures and images for: Clinical outcomes and treatments effectiveness in status epilepticus resolved by antiepileptic drugs: A five‐year observational study
Source: Epilepsia Open. 2020 Mar 2;5(2):166–75. doi: 10.1002/epi4.12383 (PMC7278543; doi:10.1002/epi4.12383)

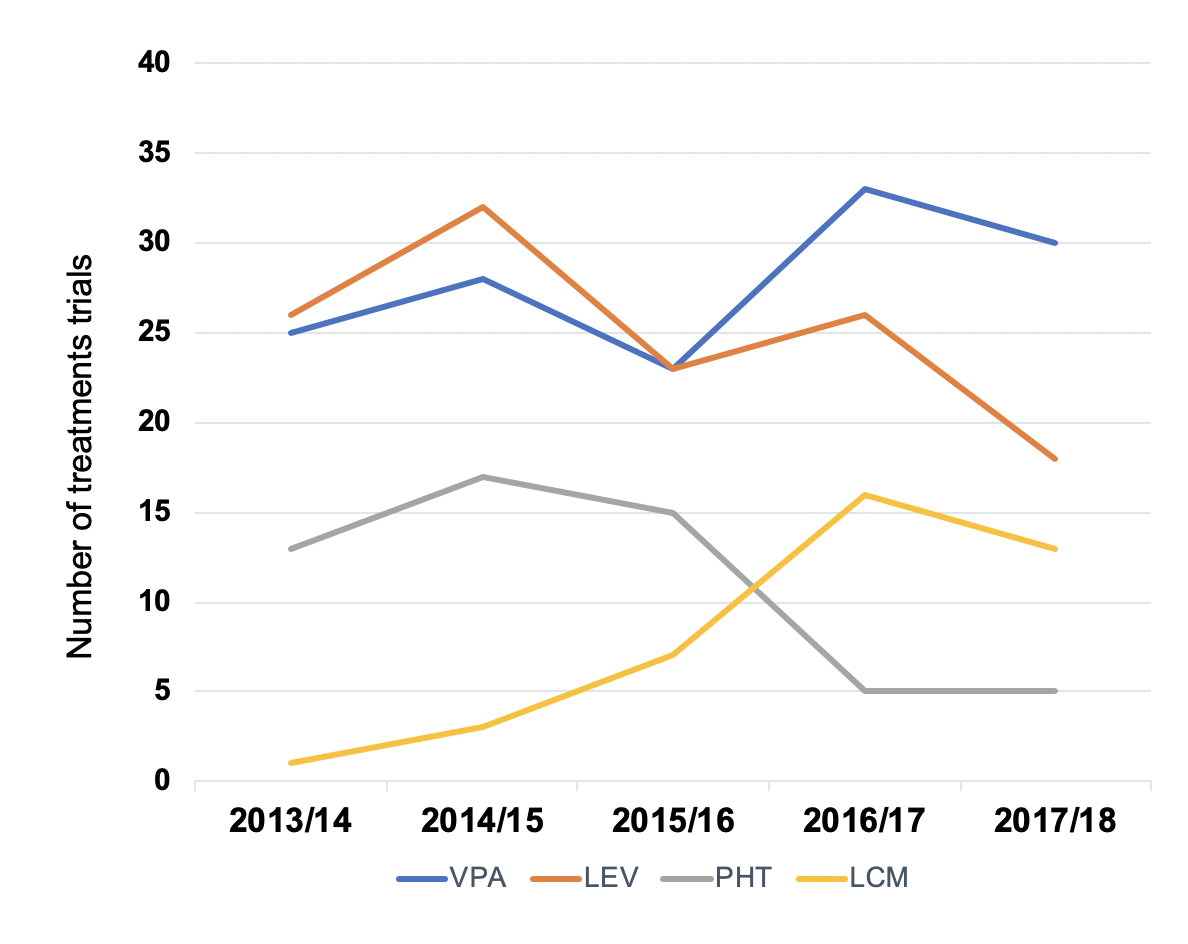

Supplement: Supplementary file 1 — FigureS1 [file EPI4-5-166-s001.tiff]
