# Supplementary material for: Clinical outcomes and treatments effectiveness in status epilepticus resolved by antiepileptic drugs: A five‐year observational study
Source: Epilepsia Open. 2020 Mar 2;5(2):166–75. doi: 10.1002/epi4.12383 (PMC7278543; doi:10.1002/epi4.12383)
Supplement: Supplementary file 2 — TableS1 [file EPI4-5-166-s002.docx]

**Online supplementary Table 1.**

Benzodiazepine and antiepileptic drugs doses (bolus and maintenance) used during the study period.

| **Drugs** | **Dosage** |
| --- | --- |
| **Lorazepam** | 0.1 mg/Kg (up to 4 mg), diluted in 10 cc of saline solution (SS), IV bolus (maximum rate 2 mg/min). If necessary it can be repeated once after 5-10 minutes. |
| **Diazepam** | 0.15 mg/Kg (up to 10 mg), diluted in 10 cc of SS, IV bolus (maximum rate 5 mg/min). If necessary it can be repeated once after 5 minutes. |
| **Midazolam** | 0.2 mg/Kg IM (up 10 mg or 5 mg in case of elderly patients and low body weight (<40 Kg). |
| **Valproate** | 20-40 mg/kg IV rapid loading dose at maximum rate 3-6 mg/Kg/h. Maintenance dose at 1-2 mg/Kg/h in 50 cc of SS in pump syringe. |
| **Phenytoin** | 20 mg/Kg IV rapid loading dose at maximum rate 50 mg/min. Maintenance dose 250 IV mg every 8 hours. |
| **Levetiracetam** | 30-60 mg/Kg (up to 3000 mg) IV rapid loading dose at maximum rate 2-5 mg/Kg/min. Maintenance dose 1000 mg IV every 8 hours. |
| **Lacosamide** | 200-400 mg IV rapid loading dose over 15 minutes. Maintenance dose 200 mg/bid IV. |
