# Supplementary material for: Clinical outcomes and treatments effectiveness in status epilepticus resolved by antiepileptic drugs: A five‐year observational study
Source: Epilepsia Open. 2020 Mar 2;5(2):166–75. doi: 10.1002/epi4.12383 (PMC7278543; doi:10.1002/epi4.12383)
Supplement: Supplementary file 4 — TableS3 [file EPI4-5-166-s004.docx]

**Online Supplementary Table 3.** **Adverse events profiles of the different used AEDs.**

| **AEDs** | **Adverse events** | **# Events per patient** | **% AE** | |
| --- | --- | --- | --- | --- |
|  |  |  | ***Specific*** | ***Total*** |
| **VPA** | Thrombocytopenia | 3 | 2.2% | 9.4% |
|  | Liver enzyme elevation | 6 | 4.3% |  |
|  | Asymptomatic hyperammonemia | 2 | 1.4% |  |
|  | Tremors | 1 | 0.7% |  |
|  | Mental confusion | 1 | 0.7% |  |
| **LEV** | Delirium | 1 | 0.8% | 2.4% |
|  | Agitation | 1 | 0.8% |  |
|  | Mental confusion | 1 | 0.8% |  |
| **LCM** | Cerebellar Ataxia | 1 | 2.5% | 7.5% |
|  | Diplopia | 1 | 2.5% |  |
|  | Skin Rush | 1 | 2.5% |  |
| **PHT** | Cerebellar Ataxia | 1 | 1.9% | 13.2% |
|  | Thrombocytopenia | 1 | 1.9% |  |
|  | Skin Rush | 2 | 3.8% |  |
|  | Arrhythmias | 1 | 1.9% |  |
|  | Diplopia | 1 | 1.9% |  |
|  | Mental confusion | 1 | 1.9% |  |

See text for statistical comparisons between AEDs.
